# Perceptions about Probiotic Yogurt for Health and Nutrition in the Context of HIV/AIDS in Mwanza, Tanzania

**DOI:** 10.3329/jhpn.v30i1.11273

**Published:** 2012-03

**Authors:** Melissa A. Whaling, Isaac Luginaah, Gregor Reid, Sharereh Hekmat, Amardeep Thind, Joseph Mwanga, John Changalucha

**Affiliations:** ^1^Department of Geography, Schulich School of Medicine and Dentistry, University of Western Ontario, London, Ontario, Canada; ^2^Departments of Microbiology and Immunology and Surgery, Schulich School of Medicine and Dentistry, University of Western Ontario, London, Ontario, Canada; ^3^Department of Food and Nutritional Sciences, Brescia University College, University of Western Ontario, London, Ontario, Canada; ^4^Department of Family Medicine, Schulich School of Medicine and Dentistry University of Western Ontario, London, Ontario, Canada; ^5^University of Western Ontario, London, Ontario, Canada; ^6^National Institute for Medical Research, Mwanza, Tanzania

**Keywords:** Acquired immunodeficiency syndrome, Health, HIV, Nutrition, Perceptions, Probiotics, Qualitative studies, Yogurt, Tanzania

## Abstract

Recently, the food and malnutrition issues have taken centre stage within the arena of HIV/AIDS epidemic, with several calls being made for context-specific health and nutrition interventions to deal with the emerging food insecurity and malnutrition issues in settings with high burdens of HIV/AIDS. The use of probiotics as nutritional supplements in HIV/AIDS-affected and resource-poor settings has also been advocated. This paper presents the results of a qualitative study on community knowledge and perceptions about probiotics and their potential impact on people's everyday life in the context of the HIV/AIDS epidemic. In-depth interviews (n=26) were conducted with residents in Mwanza, Tanzania. The results showed that people living with HIV/AIDS, who were using probiotic yogurt produced through a joint partnership of Western Heads East, Tanzania Medical Research Institute and the Tukwamune Women's Group, reported perceived beneficial effects, such as gain in weight and improved health and well-being. Yet, these beneficial effects might be resulting in growing misconceptions about probiotic yogurt being ‘medicine’ for the treatment of HIV/AIDS; this is leading some people living with HIV/AIDS to abandon taking their antiretroviral medications based on the view that the probiotic yogurt is making them feel much better. The findings illustrate the potential challenges with regard to the introduction of nutritional food supplements into new contexts plagued by malnutrition and infectious diseases. Public-health education and awareness programmes are needed when introducing novel foods into such contexts.

## INTRODUCTION

In 2003, HIV/AIDS became the leading cause of death among adults in Tanzania. By early 2008, it was estimated that 1.3 million people, including adults and children aged less than 18 years, in Tanzania mainland were living with HIV (1). Similar to other countries in sub-Saharan Africa (SSA), the HIV/AIDS epidemic in Tanzania is predominantly spread through heterosexual relationships and influenced by a complex and interrelated set of social, economic, behavioural and biological factors. Demographically, the epidemic is also unevenly distributed, with women, young people, and the poor being more likely to be infected (2). The national rate of HIV/AIDS prevalence is estimated to be about 6.0%; yet rates up to 15% were reported in places, such as Mbeya, Iringa, and Mwanza (3). Against the backdrop of the challenges faced by the SSA governments to provide adequate HIV/AIDS and health services to their populations, increasing attention is being directed towards the diverse range of local organizations and informal groups that are forced to bear an increasing share of responsibility through direct involvement in the provision of HIV/AIDS-prevention and care services (4).

For people living with HIV/AIDS (PLWA), antiretroviral therapy improves immunity, slows down disease progression, and increases life expectancy. However, nausea, dizziness, vomiting, and contribution to a poor quality of life are often cited as reasons for non-adherence to and discontinuation of antiretrovirals (ARVs) (5,6). The intensity of side-effects of ARVs is often greater in food insecure populations where people experience micronutrient deficiencies (6). It has been recognized that deficiencies in micronutrients may lead to a decrease in individual body mass and wasting in advanced HIV patients, and it may also trigger gastrointestinal malabsorption and increased metabolic demand (7). Micronutrient deficiencies are also independently linked to low cluster of differentiation 4 (CD4) cell counts (a measure of immune status), advanced HIV-related diseases, faster disease progression, and HIV-related mortality (8,9). On the other hand, there is clear evidence that, when combined with a well-nourished diet, ARVs have a greater impact on the health and life expectancy of PLWA (5,6,10). A number of studies have suggested the applicability of nutritional and microbial supplements, such as probiotics, as complementary to ARV treatment for PLWA (11-15).

More recently, the food and malnutrition issues have taken centre stage within the arena of HIV/AIDS epidemic (16-18), with several calls being made for community-specific health and nutrition interventions that are best adapted to deal with the emerging food insecurity and malnutrition issues in settings with high burdens of HIV/AIDS. The recognition of the importance of nutrition in the transmission, treatment, and progression of HIV led to the recommendation that those infected with the virus consume adequate quantity of foods that are high in vitamins and micronutrients since these can help boost the immune system (19-22).

The Food and Agriculture Organization/World Health Organization identified probiotic foods as substances with a strong potential to provide health benefits, especially to food-insecure populations and those at a high risk of infectious diseases (20,21,23). Probiotics are defined as live microorganisms which, when administered in adequate quantity, confer a health benefit on the host. As emerging food supplements, probiotics have shown a significant impact on the health and well-being of people (24-26). Studies have shown that probiotic strains can improve host defenses and enhance the quality of life of people (27-29). It is also suggested that probiotic bacteria have the potential to destroy HIV *in vitro* and reduce the risk of bacterial vaginosis, a condition that predisposes women to sexually transmitted diseases and increases the risk of mother-to-child transmission of disease (29-32). The evidence also indicates that probiotics can boost immunity, increase CD4 cell counts, prevent diarrhoea, constipation, and urinary tract infections (11,32-36).

In response, several community nutrition programmes in the contexts of HIV/AIDS-burden have been implemented (6,13), with some programmes focusing on the development of community health. For instance, the University of Western Ontario (UWO), in collaboration with the Kivulini Women's and Children's Rights Organization (Kivulini) and the Tanzanian National Institute of Medical Research (NIMR) implemented a sustainable probiotic food-based project titled “Western Heads East” (WHE) in Mwanza, Tanzania, to improve nutrition among the PLWA. The WHE project was initiated in 2003 and currently employs local women (Yogurt Mamas) from the Tukwamuane Women's Group (TWG), who produce probiotic yogurt daily in a community kitchen. The probiotic bacterium (*Lactobacillus rhamnosus* GR-1) used in the yogurt (26) is cultured at the laboratories of the NIMR in Mwanza and is delivered to the kitchen every two weeks. The NIMR also oversees the safety and effectiveness of the probiotic yogurt culture. Local cow-milk is delivered daily to produce the yogurt. The yogurt is consumed by the Yogurt Mamas and their family members, and 200 mL/day of probiotic yogurt is given to the registered PLWA in the community and is also sold to the public. Within this larger project, the measured health impacts of the probiotic yogurt among PLWA were reported elsewhere (15,32).

There is an increasing demand for programmes involving the use of macronutrients and food supplements in the HIV/AIDS-affected regions and an emerging need to expand the existing projects to other countries. For instance, with the World Bank's funding (37), two probiotic yogurt kitchens have been established in Oyugis, Kenya. These changes need to recognize that the varying perceptions and constructions of the illness experience of the affected people help shape their behaviours and responses, including their adherence to medical regimens, such as ARVs, and their search for new forms of treatment (38). In the case of probiotic yogurt, these perceptions may also influence its acceptability and use. As such, there is a need to understand the local people's perceptions and use of probiotic yogurt and other nutritional supplements in the context of the HIV/AIDS epidemic. In response, the objective of this study was to use interpretative methods to explore community knowledge and perceptions about the probiotic yogurt and the perceived impacts on people's everyday life in the context of the HIV/AIDS epidemic.

## MATERIALS AND METHODS

### Study context

Healthcare in Tanzania is currently provided by a mixture of government, private not-for-profit and private-for-profit agencies but there are numerous issues that stem from access and quality of care. Many public facilities have shortages of staff and high rates of staff burnout, poorly-developed infrastructure, and an often-scarce supply of medicines, drugs, and equipment (39-41). The financing issues have dwindled healthcare expenditure to only US$ 8 per person per year, which meets only one-third of the healthcare needs and requirements of the public system (41). Currently, a large segment of the Tanzanian population, particularly the poor, has limited access to basic health services, and some have no access at all (42). Mahina is a relatively-poorer community in Mwanza and was chosen in collaboration with our study partners, such as Kivulini, WHE, TWG (Yogurt Mamas), NIMR, and Bugando Hospital. It is estimated that the prevalence of HIV/AIDS in this community is over 18%, which is 2-3 times higher than the national HIV/AIDS prevalence rate of 8.8% (43). Healthcare provision in the community is plagued with the poor infrastructure and lack of services. High cost of transportation has also contributed to the challenges the people face in trying to access healthcare in the urban core.

Since in-depth information was required, we employed a qualitative methodology for this study. A snowball-sampling technique was used for contacting participants. This method of data-collection enables the researcher to contact hard-to-reach respondents who are willing to discuss sensitive issues, such as those relating to HIV/AIDS (44). Participants were introduced to the study through the gatekeepers and subsequently by other community members. Interviewees were asked to suggest somebody's name (irrespective of his/her HIV/AIDS status) we might interview next, who does not necessarily share his/her views on probiotic yogurt, health, and HIV/AIDS in his/her community.

Semi-structured interviews were conducted with 26 participants (males=10, and females=16) aged 20-65 years. The interviews were guided by a checklist that was developed based on the overarching themes of knowledge and perceptions of probiotic yogurt (11,23,29,45). The interview guide consisted of open-ended questions with associated prompts and probes that expanded and elicited in-depth discussion relating to knowledge of probiotics, perceptions about health, and other study objectives. For example, the questions included: ‘Can you describe for me your knowledge about probiotics (yogurt)?’; ‘In relation to your health and well-being, can you describe your experiences since you have been eating probiotic yogurt from the Mabatini kitchen?’; ‘How do you feel when you take probiotic yogurt?’; ‘How do you feel when you take ARVs?’; and ‘Could you describe your experiences of taking the probiotic yogurt and your ARVs’?

To ensure that the interview checklist was culturally and contextually appropriate, the WHE researchers and members of Kivulini, who have significant knowledge of the community, reviewed the interview guide before making contact with the participants. The interview checklist was also tested with members of the Mabatini community (where the current probiotic yogurt project is located) to ensure that the questions would be acceptable to the community. The feedback from test participants was taken into account, and the necessary changes were made to the interview instrument.

The interviews, on average, lasted for approximately 45 minutes and were translated by a trained research assistant from the St. Augustine University in Mwanza from English to Swahili and back to English. Interviews were conducted either at the local community centre or at the participants’ homes to ensure the comfort of the participants. All the tape-recorded interviews were transcribed verbatim for analysis.

### Analysis

The mode of analysis of the interviews was guided by the literature on conceptions and perceptions of probiotics, nutritional supplements, and HIV/AIDS (46-48). Each transcript was examined line-by-line using the QSR NVivo qualitative software. Each line and paragraph of text was thematically coded using categories of responses to the questions that were developed before coding. Emerging theme codes were also added as they appeared. The major theme categories were reviewed numerous times to ensure that classification was adequate. Thematic coding allowed the researcher to interpret and analyze data from the ground up. The significance of categorization was based upon qualitative connections, relationships, word association, and modelling. This inductive approach was used for allowing data to direct the progression of the intended and emergent themes, including perceptions about probiotic yogurt for health and nutrition.

In each segment of the data representation, we provide quotations from the data. The participant's name, represented by a pseudonym and his/her sex, are provided at the end of each quotation.

### Ethical approval

Ethical approval for the study was obtained from both UWO Research and NIMR Ethics Boards. Initial contacts with the community leaders (gatekeepers as they are called in the context) were made through our partners. Meetings were then held with the community chairperson and gatekeepers to discuss the research objectives and to secure their support and approval of the study. Before interviews, the participants were informed that their participation was voluntary and they may withdraw from the interview anytime. 

## RESULTS

The majority (61%) of the 26 participants were married (Table 1) and were aged 21-63 years. Their average age was 35 years. All the participants had children, with an average number of four children per family, which is comparable with the national average of 5.11 children (49). The themes that emerged during the coding process included: knowledge of probiotics, perceived health benefits of probiotic yogurt, probiotic yogurt as ‘medicine’, and abandonment of ARVs.

**Table 1.** Sample characteristics

Pseudonym	Sex	Marital status	HIV status
Anisia	F	Widowed	+
Beatrice	F	Widowed	-
Maiden	F	Married	+
Nitma	F	Widowed	+
Nadia	F	Separated	+
Sadiki	F	Widowed	+
Mwaka	M	Married	-
Afifa	F	Widowed	+
Durhame	F	Married	+
Shamim	M	Married	-
Kirstin	F	Separated	-
Betoto	M	Married	-
Sylvia	F	Married	+
Duke	M	Married	+
Stephan	M	Married	-
Malia	F	Married	+
Aziza	F	Single	+
Lambardo	M	Married	+
Bahati	M	Married	+
Lobbo	M	Married	+
Mossitti	M	Married	+
Safi	M	Married	+
Bahiya	F	Married	+
Siri	F	Married	+
Rabina	F	Widowed	+
Merin	F	Widowed	+

+=Positive;

−=Negative;

F=Female;

M=Male

### Knowledge of probiotics

Twenty-one participants reported that they had eaten the probiotic yogurt produced by the TWG. Seventeen consumed the probiotic yogurt at least three times per week. Thirteen had knowledge regarding the health benefits of probiotics. Overall, the participants indicated that their knowledge and understandings of probiotics were drawn from lay-people (Yogurt Mamas and other yogurt consumers) and from professionals, such as doctors and nurses, at the Sekou Tuore and Bugando Hospitals, who sometimes referred HIV/AIDS patients to the probiotic yogurt kitchen. Other sources of the information included ARV clinic staff, local organizations, and non-profit organizations, such as Kivulini and the Tanzanian Commission for AIDS (TACAIDS).

Most (81%) participants who were eating the probiotic yogurt agreed that their knowledge about the yogurt was limited. In the following statement, a participant explained that she regularly consumed the probiotic yogurt but did not know anything about its special properties:

… I take the [probiotic] yogurt about 4 or 5 times a week. I like its taste. But I must tell you, I do not know what is in the yogurt. I was told that I could register to get it [the probiotic yogurt] free of charge because I have HIV…since I started eating it, I feel so much better…but I do not know why this yogurt is so good for me…I just do not know what is put in there and why it helps…. I can cook milk at home but it does not help me feel the same way (Merin, female, HIV+ve).

Those who had more in-depth knowledge (Table 2) identified that probiotics are bacteria (*bakteria* in kishwahili language) that benefit health. The following quote describes one woman's perception about the probiotic bacteria:

I do not know everything [about the probiotic yogurt] but I do know that it has good *bakteria* in it and that make you healthy. The doctor at the clinic said something about how *bakteria* are not always bad and the probiotic yogurt has the good *bakteria* in it that make us healthy (Maiden, Female, HIV+ve).

The lack of knowledge on probiotics did not limit the participants’ overall perceptions about the health benefits of probiotic yogurt.

### Perceived health benefits of probiotic yogurt

The participants described several perceived benefits of probiotic yogurt to their health and well-being. For instance, in the comment below, Lobbo reported that the consumption of probiotic yogurt boosts his energy and makes him feel ‘stronger’:

It [the probiotic yogurt] makes me feel stronger and gives me energy. Before I used to feel so tired, and I did not want to do anything but since I started taking the yogurt, I noticed that my energy is back…. I feel strong again (Lobbo, male, HIV+ve).

Other participants specifically focused on the benefits for the PLWA and talked about the potential of probiotics to increase immunity, including the CD4 counts of PLWA who are taking the yogurt:

People with HIV are the ones taking it daily…. It is good for everyone but for people like me with HIV, it really helps. It makes the immune system work better and has made my CD4 count go up to 500. I take ARVs and these help too but with the [probiotic] yogurt it works better (Sylvia, female, HIV+ve).

I was told that it would be beneficial for me to take the yogurt with my ARVs.…But the ARVs make me feel very sick sometimes, and the yogurt helps stop this…it makes me feel better. It is good for those of us with HIV. It increased my CD4 count, and now I am healthier. I think that it makes the ARVs work better too…that is why I take it (Bahati, male, HIV+ve).

Among other nutritional benefits, the participants talked at length about how consuming probiotic yogurt causes gain in needed weight:

I know this is good because I have gained weight …this is what I needed because my clothes were starting to fall off from me….HIV/AIDS makes you lose weight, and this is not good for you (Durhame, female, HIV+ve).

My friend who eats the yogurt has gained weight from eating it, which is good because she was so skinny. You can see it in her body and face that this is good for her…she looks healthier since she has been taking the yogurt (Kirstin, female, HIV-ve).

Some respondents also talked about the impact of the probiotic yogurt on their gastrointestinal health by reporting that, since they started taking the yogurt, they experienced fewer stomach upsets and pain.

When I take probiotic yogurt, I do not get stomach upsets anymore. Before my stomach would really hurt … especially with ARVs … my stomach would churn inside, and I would get a terrible pain in the lower abdomen. Now, this does not happen so much…. (Duke, male, HIV+ve).

It [the probiotic yogurt] stops the pain in my stomach…. It used to hurt so much…and all the time, it just did not feel right. Now, it is a lot better…so I go about my daily activities more freely (Lobbo, male, HIV+ve).

The reduction of diarrhoeal episodes as a result of eating probiotic yogurt was also reported by some participants. One participant noted that she now sees the doctor less often because she does not experience many diarrhoeal episodes:

… this [the consumption of probiotic yogurt] has helped make my diarrhoea happen less often. I used to go to the clinic a lot more because of diarrhoea, and now I only have to go once in a while (Nitma, female, HIV+ve).

The participants also mentioned other health effects of probiotic yogurt, including the ‘clearance of skin rashes’ and ‘reduced pain in lower limbs’:

Well, I used to have rashes on my skin that would not go away but since I have started taking probiotic yogurt, it has almost all gone away. See this spot of rash here [points to arm] …this used to be all over my body and now it is not. The [probiotic] yogurt has cleared it up (Lambardo, male, HIV+ve).

For some reason, my legs have stopped hurting as much. I used to get such bad pain in the muscle and joints, and now that I am eating the [probiotic] yogurt, this is no longer the case (Sylvia, Female, HIV+ve).

While the participants talked about the benefits of the probiotic yogurt, it also emerged in the study that 38% of the participants viewed probiotic yogurt as medicine and not just a nutritional supplement.

### Probiotic yogurt as ‘medicine’ for treating HIV/AIDS: a misconception

In the following passages, the respondents conceptualized probiotic yogurt as ‘medicine’ for treating HIV/AIDS, thereby highlighting a misconception:

… Now, I take the yogurt as medicine for my HIV/AIDS and lots of other things like diarrhoea and stuff like that. This medicine is making me feel good…. It is something that helps so much, and there are no side-effects when I take it (Mossitti, male, HIV+ve).

… people take it as medicine…it cures everything. Some people think that it is a miracle medicine because it is just much better than the ARVs…it does not make people feel sick, it makes me feel healthy.…I would like to know and understand what exactly the probiotic yogurt does for people who are HIV-positive… (Nitma, female, HIV+ve).

While the overall health impacts of probiotic yogurt are acknowledged in the literature, the problem whereby some HIV/AIDS patients are abandoning their ARVs because they are eating probiotic yogurt and perceiving it as ‘medicine’ is a concern.

### Abandoning of ARVs

In the following excerpt, a respondent explained why she stopped taking her ARVs:

… I noticed myself, from taking the yogurt, that I felt really good. I now have energy I did not have before…. I feel strong now. So, I know that this probiotic medicine is something that is good for my health. This is why I stopped taking ARVs. The yogurt is helping me so much that I do not even have to take ARVs. This is good because they [ARVs] made me feel very sick before, and now I have energy. I also like the taste of the yogurt. It is a good meal for me (Anisia, female, HIV+ve).

Similarly, another respondent commented on the effectiveness of probiotic yogurt and how it has resulted in an increase in her CD4 count.

Probiotic yogurt is a powerful medicine … it just makes me feel healthy. I take yogurt instead of ARVs to help me feel better from the HIV. The ARVs used to make me feel very sick, and I do not like them. Also, since I have been taking the yogurt, my CD4 counts have gone up, and this is why I know that it helps my immune system. My CD4 count has gone from 235 to 315. Since this is helping me so much, I stopped taking my ARVs.… It is simple, the ARVs make me feel sick but the yogurt does not, and it tastes good (Durhame, female, HIV+ve).

As demonstrated in the above quotations, the abandonment of ARVs in place of probiotic yogurt suggests a lack of understanding of the role of ARVs to PLWA, and the fact that probiotic yogurt is only a nutritional supplement. A summary of the interview responses are provided in Table 2.

## DISCUSSION

The HIV/AIDS epidemic continues to present unique and severe challenges for the global health community, especially in the hardest-hit areas, such as SSA. Countries, such as Tanzania, are impacted in multidimensional ways, and the effects are exacerbated by chronic poverty, growing food insecurity, and severe malnutrition. As the local governments in SSA make attempts to provide ARVs to those infected with HIV, it is imperative that the nutritional status of PLWA is kept in mind since well-nourished individuals who use ARVs have reduced side-effects and are more likely to live longer.

The importance of nutrition in both transmission and progression of HIV led to the recommendation that those infected should consume adequate quantity of foods that are high in vitamins and micronutrients and can help boost the immune system (20-22). The WHE project that formed the basis of this research offers such a unique community-based health and nutrition intervention that focuses on community development and the use of local ideas, resources, skills, and knowledge for better health in the context. The participants’ descriptions of the perceived benefits of probiotic yogurt were consistent with the literature on probiotic food supplements, including gain in weight (14,15) and increases in CD4 counts ( ,15,29,36). It is well-known that probiotics play a key role in the regulation of gastrointestinal health (27,50) and reduce diarrhoea (36,51). Furthermore, the reduction of pain in the lower limbs of some respondents in this study could be attributed to the advanced absorption of micronutrients and immunity-boosting properties of probiotics (32-35).

Despite the positive therapeutic benefits of probiotics, the misconception by some participants that probiotic yogurt is a ‘medicine’ for the treatment of HIV/AIDS emerged as a major concern. Understandably, those who were referring to probiotic yogurt as ‘medicine’ may have been using the term in relation to their individual perceptions and cultural constructs of health and well-being embedded in the context, to the extent that what makes people 'feel better’ becomes their ‘medicine’ (52). As the individual's everyday life-circumstances and experiences often vary, so do the perceptions and meanings they attach to health, illness, medicine, and any other available treatment options (53-56). These systems of meaning also influence expectations and perceptions of symptoms, and the responses to sickness and disease (53).

Most indigenous approaches to healing are principally concerned with treating illness as the human experience of sickness rather than the biological recognition and treatment of disease (48,57). Consequently, people with chronic illnesses, such as HIV/AIDS, in resource-poor settings, who spend most of their time away from medical environments, may rely on their own perceptions, beliefs, knowledge, judgements, and resources for managing their illness (58).

**Table 2.** Summary of responses from in-depth interviews

Response	No. mentions	Participants	
		No.	%
Knowledge of probiotics			
Awareness of Mabatini probiotic yogurt kitchen	23	23	88
Ever-eaten probiotic yogurt	21	21	81
Probiotic yogurt contains *bakteria* (bacteria)	15	12	46
Lack of any knowledge	11	5	9
Desire to learn and know more about probiotics	15	9	35
Perceived health benefits of probiotic yogurt			
Increases CD4 counts	11	8	31
Boosts or gives energy	37	20	77
Makes one feel stronger	45	16	62
Gain in weight	23	11	42
Makes ARVs work better	9	6	23
Taken probiotic cleared my rashes	7	3	12
Prevents stomach upsets	21	9	35
Reduces diarrhoea	13	7	27
Probiotic as medicine	25	10	38
No side-effects	11	5	19
ARVs			
ARVs make me feel sick	18	13	50
Since I have been taking probiotic yogurt, I stopped taking my ARVs	9	4	15
I do not like ARVs	14	9	35

ARVs=Antiretrovirals

Within the context of the HIV/AIDS epidemic in SSA, the increasing availability of several nutritional supplements and therapies means that individual's decisions about their preferred options will frequently revolve around what makes them feel better. Some people in this category then stop taking their ARVs with the belief and hope that the probiotic yogurt is the best option for their better health and well-being. This is consistent with the literature whereby patients with non-curative diseases, such as HIV/AIDS, may be so desperate such that any treatment, medicine, procedure, or substance that make them feel better or minimize their symptoms become the best therapy for them, regardless of biological pathways and markers that may indicate otherwise (59). To a person living with HIV/AIDS, anything that helps them cope with their everyday activities, or anything that gives them hope for a longer life will become a significant add-on to thequality of their lives (60-62).

While we acknowledge the therapeutic effects of probiotics, there is no scientific recommendation that they can be used for treating HIV or replace ARVs. However, results of several studies suggest that the combination of ARVs with probiotics is, in fact, beneficial to the patient due to the numerous therapeutic and nutritional properties of probiotic yogurt (7,12,13,21). The view is that ARVs can often kill off beneficial intestinal flora, allowing the overgrowth of harmful bacteria that cause diarrhoea. It has been shown that such side-effects from taking ARVs can be reduced or even be reversed by consuming yogurt with live probiotic cultures. Hence, given the misconceptions about probiotics and other nutritional supplements as treatments for HIVAIDS, these settings signal that community education on the benefits of nutritional supplements must receive appropriate attention where new programmes are being implemented.

### Limitations

This study has a number of limitations that must be acknowledged. First, with regard to the context in which the study was done, there is a possibility that interview responses were distorted by personal biases, communal motivations, or emotional states. Second, it is also possible that, given the nature of the topic, the responses were made out to be self-serving. For instance, the participants may have over-exaggerated or downplayed certain issues because they thought that it would have some impact on whether or not a current probiotic yogurt kitchen will continue to serve their needs or whether a new kitchen will be set up in their neighbourhood. Consequently, while the information gathered here will be useful in SSA and other HIV/AIDS-affected regions, the pronounced contextual differences in various regions have to be kept in mind when these findings are being generalized too broadly.

### Conclusions

With regard to the increasing availability (and promises of better health) of probiotics and other nutritional supplements to benefit the health of the general population, policy-makers and community workers need to be forthcoming with explicit information regarding what nutritional supplements, such as probiotics, can do or cannot do, more so that these supplements are not to replace ARVs as treatment for HIV/AIDS.

## ACKNOWLEDGEMENTS

The authors thank the community leaders and people of Mahina, Mwanza, Tanzania, for participating in the study. Special thanks are due to the Tukwamuane Women's Group ‘Yogurt Mamas’ who have been providing probiotic yogurt to community members. The authors also thank everybody involved with Western Heads East for their relentless efforts with the entire probiotic yogurt programme in East Africa. They also thank the anonymous reviewers for their extremely-useful comments and suggestions.

## References

[1] Tanzania Commission for AIDS Tanzania—2010 country progress report. Submitted to the UN General Assembly. 45 p. (http://www.unaids.org/en/dataanalysis/monitoringcountryprogress/2010progressreportssubmittedbycountries/tanzania_2010_country_progress_report_en.pdf, accessed on 24 January 2011).

[2] Joint United Nations Programme on HIV and AIDS (2008). Tanzania country profile 2008.

[3] Tanzania (2006). Prime Minister's Office. Tanzania Commission for AIDS.

[4] Luginaah I, Maticka-Tyndale E, Kairi W, Wildish J, Brouillard-Coyle C (2007). Extending HIV/AIDS-prevention efforts in Kenya: primary schools as community-based organizations. Environ Plann C.

[5] Hogg RS, Heath KV, Yip B, Craib KJP, O'shaughnessy VO, Schechter MT (1998). Improved survival among HIV-infected individuals following initiation of antiretroviral therapy. JAMA.

[6] Tomkins AM (2005). Evidence-based nutrition interventions for the control of HIV/AIDS. S Afr J Clin Nutr.

[7] Drain PK, Kupka R, Mugusi F, Fawzi WW (2007). Micronutrients in HIV-positive persons receiving highly active antiretroviral therapy. Am J Clin Nutr.

[8] Bogden JD, Baker H, Frank O, Perez F, Kemp F, Bruening K, Louria D (1990). Micronutrient status and human immunodeficiency virus (HIV) infection. Ann N Y Acad Sci.

[9] Nerad J, Romeyn M, Silverman A, Allen-Reid J, Dieterich D, Merchant J (2003). General nutrition management in patients infected with human immunodeficiency virus. Clin Inf Dis.

[10] Paton N, Sangeetha S, Earnest A, Bellamy DR (2006). The impact of malnutrition on survival and the CD4 count response in HIV-infected patients starting antiretroviral therapy. HIV Med.

[11] Reid G (1999). The scientific basis for probiotic strains of *Lactobacillus*. Appl Environ Microbiol.

[12] Foster HD (2007). New strategies for reversing viral pandemics: the role of nutrition. Proceedings of the International Forum for Public Health.

[13] Namulemia E, Sparling J, Foster HD (2007). Nutritional supplements can delay the progression of AIDS in HIV-infected patients: results from a double-blinded, clinical trial at Mengo Hospital, Kampala, Uganda. J Orthomol Med.

[14] Trois L, Cardoso EM, Miura E (2008). Use of probiotics in HIV-infected children: a randomized double-blind controlled study. J Trop Pediatr.

[15] Irvine SL, Hummelen R, Hekmat S, Looman CWN, Habbema JD, Reid G (2010). Probiotic yogurt consumption is associated with an increase of CD4 count among people living with HIV/AIDS. J Clin Gastroenterol.

[16] Gillespie S, Kadiyala S (2005). HIV/AIDS, food and nutrition security: from evidence to action.

[17] Anabwani G, Navario P (2005). Nutrition and HIV/AIDS in sub-Saharan Africa: an overview. Nutrition.

[18] Semba RD, Tang AM (1999). Micronutrients and the pathogenesis of human immunodeficiency virus infection. Br J Nutr.

[19] Food and Agriculture Organization (2001). Health and nutritional properties of probiotics in food including powder milk with live lactic acid bacteria.

[20] Food and Agriculture Organization (2002). Guidelines for the evaluation of probiotics in food. Joint FAO/WHO Working Group report on drafting guidelines for the evaluation of probiotics in food.

[21] Food and Agriculture Organization (2006). Probiotics in food: health and nutritional properties and guidelines for evaluation.

[22] Haddad L, Gillespie S (2001). Effective food and nutrition policy responses to HIV/AIDS: what we know and what we need to know. J Int Dev.

[23] Reid G, Jass J, Sebulsky T, McCormick JK (2003). Potential uses of probiotics in clinical practice. Clin Microbiol Rev.

[24] Lorea Baroja M, Kirjavainen PV, Hekmat S, Reid G (2007). Anti-inflammatory effects of probiotic yogurt in inflammatory bowel disease patients. Clin Exp Immunol.

[25] Floch MH, Walker WA, Guandalini S, Hibberd P, Gorbach S, Surawicz C (2008). Recommendations for probiotic use–2008. J Clin Gastroenterol.

[26] Hekmat S, Hemsworth J, Reid G, Soltani H, Gough B (2008). The effects of probiotic yogurt consumption on nutritional status in Mwanza, Tanzania. J Complement Integr Med.

[27] Rolfe RD (2000). The role of probiotic cultures in the control of gastrointestinal health. J Nutr.

[28] Hummelen R, Hemsworth J, Reid G (2010). Micronutrients, N-acetyl cysteine, probiotics and prebiotics: a review of the effectiveness in reducing HIV progression. Nutrients.

[29] Lenoir-Wijnkoop I, Sanders ME, Cabana MD, Caglar E, Corthier G, Rayes N (2007). Probiotic and prebiotic influence beyond the intestinal tract. Nutr Rev.

[30] Fuller R (1991). Probiotics in human medicine. Gut.

[31] Cribby S, Taylor M, Reid G (2008). Vaginal microbiota and the use of probiotics. Interdiscip Perspect Infect Dis.

[32] Hummelen R, Hemsworth J, Reid G (2010). Feeding immunity: micronutrients and functional food components to combat HIV progression. Nutrients.

[33] Dugas B, Mercenier A, Lenoir-Wijnkoop I, Arnaud C, Dugas N, Postaire E (1999). Immunity and probiotics. Immunol Today.

[34] de Roos NM, Katan MB (2000). Effects of probiotic bacteria on diarrhoea, lipid metabolism, and carcinogenesis: a review of papers published between 1988 and 1998. Am J Clin Nutr.

[35] Blum S, Haller D, Pfiefer A, Schiffrin EJ (2002). Probiotics and immune response. Clin Rev Allergy Immunol.

[36] Anukam KC, Osazuwa EO, Osadolor HB, Bruce AW, Reid G (2008). Yogurt containing probiotic *Lactobacilus rhamnosus* GR-1 and *Lactobacillus reuteri* RC-14 helps resolve moderate diarrhea and increases CD4 count in HIV/AIDS patients. J Clin Gastroenterol.

[37] World Bank (2007). Probiotic yogurt for health and nutrition in East Africa: women helping women.

[38] Petrie KJ, Weinman J (1997). Perceptions of health and illness: current research and applications.

[39] Peabody JW (1996). Economic reform and health sector poli-cy: lessons from structural adjustment programs. Soc Sci Med.

[40] Sekwat A (2003). Health financing reform in sub-Saharan Africa: major constraints, goals and strategies. J Health Care Finance.

[41] Shiner A (2003). Shaping health care in Tanzania─who's pulling the strings?. Lancet.

[42] Kopoka A (2002). Provision of health services in Tanzania in the twenty first century: lessons from the past.

[43] Tanzania Government of The national website of the United Republic of Tanzania. (http://www.tanzania.go.tz, accessed on 5 April 2008).

[44] Faugier J, Sargeant M (1997). Sampling hard to reach populations. J Adv Nurs.

[45] Lourens-Hattingh A, Viljoen BC (2002). Yogurt as probiotic carrier food. Int Dairy J.

[46] van der Geest S (1997). Is there a role for traditional medicine in basic health services in Africa? A plea for a community perspective. Trop Med Int Health.

[47] Bryant J, Browne A, Barton S, Zumbo B (2002). Access to health care: social determinants of preventive cancer screening use in northern British Columbia. Soc Indic Res.

[48] Green M (2004). Public reform and the privatization of poverty: some institutional determinants of health seeking behaviour in southern Tanzania. Cult Med Psychiat.

[49] Olenick I (1998). In Tanzania, ideal family size closely resembles actual number of children. Int Fam Plan Pers-pec.

[50] Reid G (2008). Probiotic lactobacilli for urogenital health in women. J Clin Gastroenterol.

[51] Allen SJ Okoko B Martinez E Gregorio G Dans LF Probiotics for treating infectious diarrhoea. Cochrane Database Syst Rev 2004: Art no. CD003048. 10.1002/14651858.CD003048.pub2 15106189

[52] Kearns RA, Gesler WM (1998). Putting health into place: landscape, identity, and well-being.

[53] Kleinman A, Eisenberg L, Good B (1979). Culture, illness, and care: clinical lessons from anthropologic and cross-cultural research. Ann Intern Med.

[54] Brown CM, Segal R (1997). The effects of health and treatment perceptions on the use of prescribed medication and home remedies among African American and White American hypertensives. Soc Sci Med.

[55] Erwin J, Peters B (1999). Treatment issues for HIV + Africans in London. Soc Sci Med.

[56] Vermeire E, Hearnshaw H, Van Royen P, Denekens J (2001). Patient adherence to treatment: three decades of research: a comprehensive review. J Clin Pharm Ther.

[57] Gesler MC, Msuya D, Nkunya MH, Schar A, Heinrich M, Tanner M (1995). Traditional healers in Tanzania: the perception of malaria and it's causes. J Ethnopharmacol.

[58] Roberson MHB (1992). The meaning of compliance: patient perspectives. Qual Health Res.

[59] Duggan J, Peterson WS, Schutz M, Khuder S, Charkra-borty J (2001). Use of complementary and alternative therapies in HIV-infected patients. AIDS Patient Care STDS.

[60] Pawluch D, Cain R, Gillett J (2000). Lay constructions of HIV and complementary therapy use. Soc Sci Med.

[61] Kylma J (2005). Dynamics of hope in adults living with HIV/AIDS: a substantive theory. J Adv Nurs.

[62] Harris G, Larson D (2008). Understanding hope in the face of an HIV diagnosis and highrisk behaviors. J Health Psychol.

